# A mixed-methods process evaluation of the feasibility and acceptability of involving community and peer role models within a physical activity intervention for primary-school-aged girls (the CHARMING study)

**DOI:** 10.1186/s12889-023-16826-x

**Published:** 2023-10-07

**Authors:** Kelly Morgan, Jordan Van Godwin, Rebecca Cannings-John, Britt Hallingberg, Graham Moore, Bethan Pell, Holly Whiteley, Jemma Hawkins

**Affiliations:** 1 Centre for Development, Evaluation, Complexity and Implementation in Public Health Improvement (DECIPHer), Cardiff University, Spark, Maindy Road, Cardiff, CF24 4HQ UK; 2 Centre for Trials Research, Cardiff University, Heath Park, Cardiff, CF14 4YS UK; 3 Cardiff School of Sport and Health Sciences, Cardiff Metropolitan University, Cardiff, CF5 2YB UK; 4 Centre for Health Economics and Medicines Evaluation, Bangor University, Holyhead Road, Normal SiteBangor, LL57 2PZ Gwynedd UK

**Keywords:** Physical activity, Intervention, School, Role models

## Abstract

**Background:**

Role models have been identified as a potential means to tackle the persisting low levels of physical activity among young girls. The aim of this research was to explore the involvement of community- and peer role models within the CHARMING (CHoosing Active Role Models to INspire Girls) intervention, an intervention which aims to increase and sustain physical activity among 9–10-year-old girls. The research questions were, is it feasible and acceptable to recruit role models? and what are the perceived barriers and facilitators to the inclusion of peer role models within the intervention?

**Methods:**

A mixed methods process evaluation was embedded within a larger feasibility study, involving three secondary schools and four adjoining primary schools in South Wales, United Kingdom. One-to-one interviews were conducted with teachers (*N* = 10) across the seven schools and community role models (*N* = 10). Focus groups were conducted with 18 peer role models (older girls from adjoining secondary schools) and 18 girls aged 9–10-years who had participated in the intervention. Primary school teachers kept observation logs of each intervention session. A researcher completed observation logs of two random sessions per school. Qualitative data were analysed using thematic analysis with a combined deductive and inductive coding approach. Observation data were analysed using descriptive statistics. Data were triangulated and comparative analyses conducted across schools.

**Results:**

Twenty-three peer role models (aged 12–16-years) and 16 community role models participated in intervention delivery. Overall, the inclusion of both types of role models was shown as acceptable and feasible within the CHARMING intervention. Observation data highlighted key areas (i.e., intervention components delivered inconsistently) for further qualitative exploration. Six themes were identified during analyses; reach and access, communication, logistics, existing systems, interpersonal relationships, and perceived impacts. Themes were intertwined across the barriers and facilitators of recruitment and implementation. Areas for future improvement were highlighted.

**Conclusions:**

Findings can be used to optimise the CHARMING intervention and inform wider interventions or policies employing several role models across settings to promote physical activity among children.

**Supplementary Information:**

The online version contains supplementary material available at 10.1186/s12889-023-16826-x.

## Background

It is well known that disparities in boys’ and girls’ physical activity levels begin at an early age and persist into adolescence [1]. These disparities extend into key areas of physical activity [2] and elite sport [3], where there is an underrepresentation of girls and women [4] and children’s exposure to narrow gender norms around activities for girls versus boys can shape attitudes into adulthood [5]. This lack of participation can, in turn, impact on health and wellbeing throughout the life course. In response, and to address these disparities, there have been calls to improve girls’ physical activity levels through use of role modelling [6]. A role model can be defined as someone who influences an individual through exemplar behaviours [7]. In recent years, there has been a movement to make female role models more visible [8–10], with example role model interventions in the fields of education [11–13], physical activity [14–16] and health [17].

Typically, physical activity role model programs for females have focused on later teenage years or adult populations [14–16], thereby missing the opportunity to intervene during a peak transformational period, namely adolescence (ages 10–19 years). This latter period presents an important phase for school-based interventions, during which preventative efforts can be implemented as children (aged 10–11 years) transition from primary to secondary school [18]. During this transition period, mixed feelings of excitement and anxiety have been reported [19] as well as declines in after-school moderate-vigorous physical activity [20], and overall physical activity, and increases in sedentary behaviours [21]. Changes to the school system (e.g., environment, Physical Education curriculum and transport modes) are thought to be some of the contributing factors towards declining physical activity levels across this transition [22]. Interestingly however, a longitudinal study found that girls reported greater encouragement to participate in sport and physical activity and better physical activity role modelling upon entering secondary school [21]. As such, authors emphasise the need to create supportive environments including the visibility of role models (lead students and staff) to combat the declines in physical activity during transition [21].

A review of 11 role model programmes concluded that role models may offer a low cost and flexible approach to long-term programme delivery, offering an affordable approach to scalability [23]. The review highlighted how different groups of people can be role models to young people and that this role can function in various ways (e.g., modelling, supporting, guiding etc.). The review further highlights that the role model programmes often involve dynamic inter-relationships (e.g., altering group dynamics, evolvement of relationships over time, the context within which relationships develop etc.). Within the field of physical activity, role models are often exemplified through leadership roles, coaching others, and setting good examples of values and behaviours [24].

In terms of key characteristics of role models, MacCallum and Beltman [23] suggest that the quality of a relationship formed with a role model may be more important than matching any particular characteristics between the individual and role model. Furthermore, programmes relying on a sole role model are likely to limit the relatability and scalability of programmes. In line with wider studies demonstrating the influence of peers and friends on children’s and adolescents physical activity levels [25–27], the review highlights the importance of involving peer role models, with positive peer interactions deemed a valuable element for successful programmes [23]. The inclusion of peers as role models is thought to offer not only the opportunity to model skills and behaviours, but to also provide support among similar interests or difficulties and broaden friendship networks [23]. In Australia, peer support programmes, whereby older peers act as role models for younger students, are viewed as a way to support younger students’ school transition and skill development while developing leadership skills of older students [28, 29].

Significant importance can be attributed to understanding how best to deliver physical activity interventions involving role models. From both a national and global perspective, the World Health Organization (WHO) [6] and National Institute for Health and Care Excellence (NICE) [30] specifically recommend the use of local role models to influence physical activity levels among females and tackle gender stereotypical constructions. Existing evidence suggests that having a role model positively influences physical activity levels [31, 32], and that role model programs are most effective when there is persistent support and time to develop a long-term relationship [23, 32]. While the characteristics and importance of role models during adolescence have been established [33], this has not been tested for physical activity, particularly for pre-adolescent girls.

### Research questions

The aim of this paper is to report specifically on process evaluation findings regarding the acceptability and feasibility of involving role models (community role models and peer role models) within a school-based physical activity intervention targeting year 5 girls (aged 9–10 years). The intervention is called CHARMING (CHoosing Active Role Models to INspire Girls), it includes community role models (physical activity providers from local communities surrounding schools) and peer-role models (older girls from adjoining secondary schools), delivering different physical activity sessions taking place each week at school for a 12-week period. The intervention was designed using a participative community approach and is informed by self-determination theory (SDT) [34] and the socio-ecological model [35, 36]. The inclusion of peer role models was introduced as an intervention component informed by earlier development work [37]. A full description of the intervention has been detailed elsewhere [37, 38] and the logic model is included in Additional file 1: Appendix 1.

The research questions from the process evaluation that are addressed in this paper are:

1. Is it feasible to recruit peer role models; what refinements are required for recruitment?
2. What are perceived barriers and facilitators to recruiting role models (both community and peer)?
3. Are the role model components acceptable to all those involved?
4. What are the perceived barriers and facilitators to implementing the role model components within school-based physical activity interventions?

## Methods

### Study design

The current paper presents findings from a process evaluation which was embedded within a cluster randomised feasibility trial of the CHARMING intervention. The trial took place between January 2021 and August 2022. The main study findings are currently under review and the protocol is available as an open-access journal article [38]. The process evaluation was guided by the MRC framework [39], the specific focus of the findings reported here relate to an understanding of the role model components of the intervention and in particular the acceptability and feasibility of implementing these components as part of the intervention. We have followed the Consolidated Criteria for Reporting Qualitative Research (COREQ) checklist [40].

### School involvement

In total, seven schools across South Wales, United Kingdom, were involved in the process evaluation. This included three secondary schools, who recruited peer role models to be involved in the intervention and four feeder primary schools who participated in the intervention (pupils from primary schools would typically transition into Year 7 of the adjoining secondary school). School characteristics have been reported within the main outcomes paper (in review).

### Participants and data collection

Prior to participation, all participants were provided with a study information sheet via email or via the schoolteacher. Participants provided written informed consent or assent. We used a dual consent process for eligible girls, obtaining parent/caregiver opt-in consent and child written assent.

#### Secondary schools

Within each secondary school, a lead teacher was responsible for recruiting students to be peer role models within the intervention. Recruitment materials outlined eligible students as girls in Year groups 8–11 (aged 12–16 years), reflecting findings from earlier intervention development [38, 41]. Materials also outlined key responsibilities for peer role models which included i) taking part in sessions alongside Year 5 girls (aged 9–10 years), ii) answering questions on personal physical activity experiences and iii) promoting physical activity opportunities available in secondary school.

One-to-one semi-structured interviews with secondary school teachers (1 per school; *n* = 3) and focus groups with student peer role models (1 per school; *n* = 18 peer role models in total) explored intervention feasibility and acceptability, including factors that might have affected peer-role model recruitment, attendance, involvement and enjoyment at weekly sessions, and suggestions for improvement.

#### Primary schools

Within primary schools, lead teachers facilitated the delivery of the after-school sessions and all 9–10-year-old girls were invited to participate.

One-to-one semi-structured interviews were conducted virtually with the Head teacher at each school (three in total, as two schools shared a Head teacher) to explore the acceptability and feasibility of the intervention. One-to-one semi-structured interviews were conducted virtually with the lead primary school teachers who facilitated intervention delivery (*N* = 1 per school) to explore intervention acceptability and feasibility of implementation with regards to the involvement of role models (both community- and peer role models) alongside any suggestions for future delivery.

Focus groups were carried out with 9–10-year-old girls at each school (2 per school), one immediately after the intervention period (*n* = 18 girls in total) and 3-months later (*n* = 13 girls in total). To aid recruitment, teachers were asked to invite a group of girls who had parental consent, with group composition ideally reflecting a range of intervention attendance rates. Six focus groups took place in-person and two were conducted virtually, with group sizes ranging between three to nine girls. Discussions at each time point included an exploration of intervention acceptability and experiences of participating.

For each intervention session, primary school lead teachers were asked to complete an attendance register and 2-page observation sheet. A member of the research team also observed random sessions at each school (*N* = 8 in total) as a form of verification of teacher observation records. Observation sheets recorded whether community role models delivered the planned core components of each session fully, partially, or not at all and detail on the involvement of peer role models within and across sessions. Whilst the observation sheets provided an insight into fidelity of intervention delivery, this information also served as a point of discussion for interviews regarding feasibility and acceptability of the intervention components. For example, if a certain component was not delivered as intended, as identified in the observation sheet, interviews explored whether there were issues with the feasibility of doing this, or acceptability issues regarding that element of the intervention.

#### Community role models

Utilising online information or local authority stakeholders’ connections, individuals delivering physical activities or sports within the school’s local community were approached to participate in the intervention as community role models. Community role models (*N* = 16) were responsible for the planning and delivery of the after-school intervention sessions.

All community role models involved in intervention delivery were invited to participate in a one-to-one semi-structured interview which explored the acceptability of the intervention. Factors which might have affected recruitment and delivery were also explored alongside future recommendations.

Throughout the study, interviews and focus groups took place either virtually or face-to-face, with questions and prompts prepared within topic guides. Observations were held in person during intervention delivery. The research team undertaking data collection and analysis comprised three females (BP, KM and JH) and one male (JvG), all with prior experience and formal training in qualitative research. No prior relationship between the research team and participants was established and the participants did not know any information about the researcher collecting the data. KM and JvG were involved in the earlier study which co-produced the intervention.

### Ethics and consent

Cardiff University’s School of Social Sciences Research Ethics Committee granted ethical approval for all process evaluation components (on May 6th, 2021, SREC/4113). All participants (*n* = 83) provided written informed consent or assent. Consent from parents/caregivers of primary school children and peer role models was also provided. This included consent for publication of the findings and the use of anonymised quotations in publications.

### Analyses

Interview and focus group data were managed within NVivo v12 (QSR International Pty Ltd) software. Audio-recordings were transcribed verbatim and anonymised prior to analysis. No transcripts were returned to participants nor did any participant provide feedback on the data. A combined deductive and inductive coding approach was undertaken for thematic analysis [42], with key themes agreed by the study team. Transcripts were coded line by line, using an a priori coding scheme aligned with the research questions (informed by process evaluation guidance [39]). A third of data were double coded to check the coding scheme. Any unexpected findings identified within the data were also coded. Following completion of coding of all transcripts, each section of the coding scheme was subject to an inductive thematic analysis, in which salient themes and recurrent patterns within the data were identified and sub-themes created within each section. These sub-themes were then further interpreted to define any over-arching themes across the data. Data were analysed within participant groups initially (school staff, pupil, community role model, peer role model) and separately for each primary school-secondary school cluster (of which there were three). Following this, comparative analysis was conducted across participant groups with particular attention being paid to similarities and differences within and between participant groups and between the three clusters. One researcher (JvG) conducted the thematic and comparative analysis of all transcripts and presented draft versions to the study team for refinement. Descriptive statistics (frequencies, percentages, means, standard deviations) were used to describe observation data, which was then triangulated with the qualitative data. For example, during instances whereby quantitative data revealed issues with delivery of a role model component, data were triangulated with qualitative data to help gain a deeper understanding of the issues observed. Themes were summarised at a higher level as part of the triangulation. Throughout the results section, qualitative data and observational data are presented together.

## Results

### Sample

**Fig. 1:**
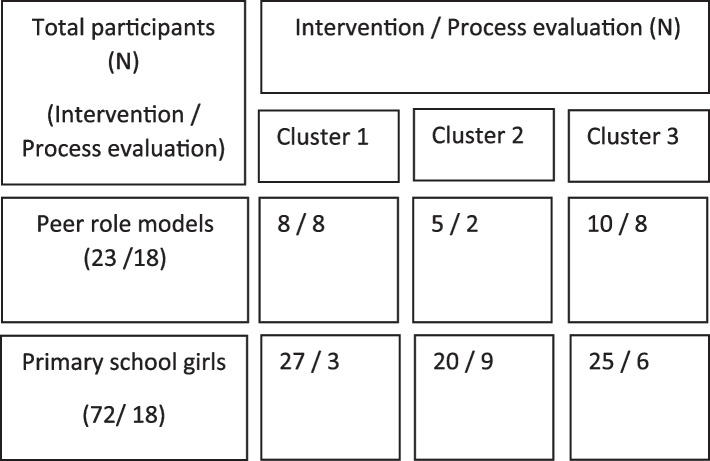
Number of year 5 girls and peer role models participating in the intervention and process evaluation

One-to-one interviews were conducted with 10 teachers across the seven schools (duration range 15–60 min) and 10 community role models (duration range 16–72 min). Six community role models did not take part in an interview (one declined and five were unavailable). Eighteen 9–10-year-old girls (of which 13 also took part at the 3-month follow-up timepoint) and 18 peer role models took part in a focus group (*n* = 11 in total), which lasted between 13–38 min and 41–54 min respectively. Figure 1 displays participation rates in the intervention and process evaluation across the three clusters. A total of 30 observation sheets (including 8 researcher forms) were completed across the 22 sessions delivered (see Table 1). Observation data are discussed alongside the qualitative data within sections below. As shown, 50% of observation forms completed by teachers had missing data, with data missing across all four schools. Reasons for the missing data are unknown.

**Table 1:**
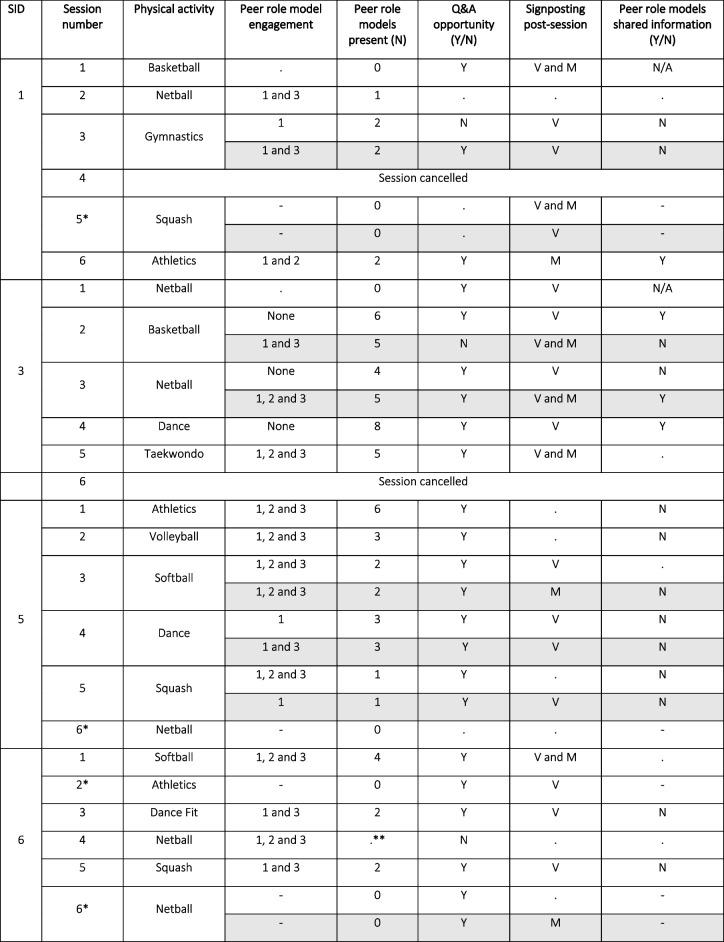
Summary of teacher and researcher observations of role model components (*N* = 30)

Grey denotes researcher observation records. *peer role models absent, **peer role models delivered the session, signifies missing data. – denotes no peer role models present. Peer role model engagement: 1 = Participating in the session, 2 = Leading parts of the session, 3 = Interacting with the Year 5 girls. Signposting: *V*  Verbal, M  Materials

**Fig. 2:**
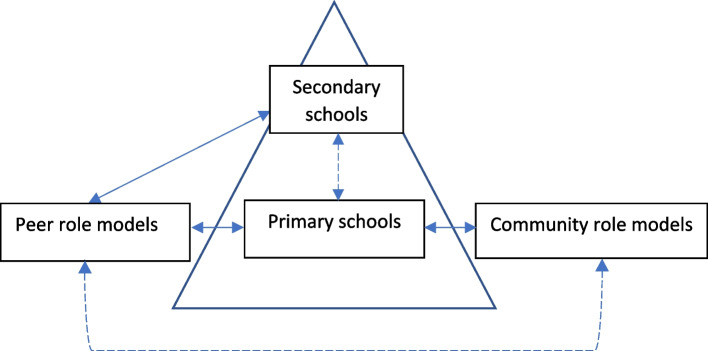
Illustration of programme communication channels (solid arrows depict clear communication channels throughout the programme whereas dotted lines signify areas where further improvements are needed)

**Fig. 3:**
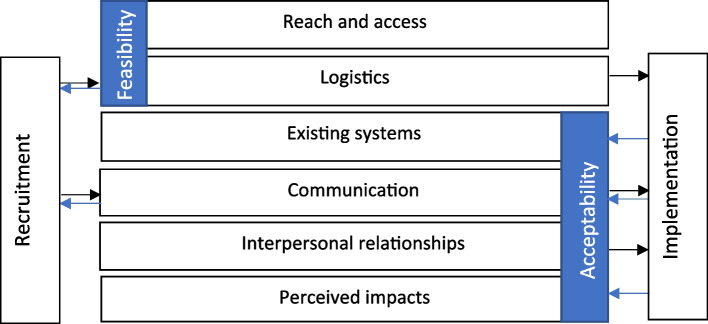
Illustration of key themes (black arrows depict barriers and blue arrows facilitators)

Six key themes were developed from analyses; reach and access, communication, interpersonal relationships, logistics, fit with existing systems and perceived impacts. Exemplar quotations are provided from a range of participants to illustrate responses across themes (Participants denoted as: GFG = Girls Focus Group, CRM = Community Role Model, PST = Primary School Teacher, SMT = Senior Management Team, SSS = Secondary School Staff, SLT = Senior Lead Teacher, with accompanying numbers showing a participant ID or group number). Figure 2 demonstrates the relationship between the role model components and schools and Fig. 3 depicts key themes. Comparative data analyses highlighted areas of differentiation across participant groups and clusters, differences are highlighted throughout the presentation of themes. The findings are presented according to the four overarching research questions.

### Research question 1: is it feasible to recruit peer role models; what refinements are required for recruitment?

Among the three secondary schools, 23 students were recruited as peer role models. Two themes were relevant to this research question, as follows.

#### Logistics

All secondary schools described the recruitment of role models as relatively easy to coordinate with limited time required to identify potential students. A consistent finding was the targeted approaches taken by all schools for recruiting peer role models. In Cluster 1, the lead teacher approached a specific year group before liaising with the physical education lead in the school to identify those students most likely to be interested in becoming peer role models in the intervention. In Cluster 2, the lead teacher approached students who were already involved in school transition work and in Cluster 3, the teacher approached a whole year group. Most students recruited as peer role models in Cluster 3 belonged to the same sports team which resulted in low peer role model attendance in one intervention session, due to a sports fixture clash. On reflection, the lead secondary school teacher highlighted the need to ensure recruitment encompassed a broad range of students to avoid this scenario. All teachers described a desire to adopt an inclusive recruitment approach in future (i.e., opening the recruitment opportunity to all eligible students), noting the constraints of COVID-19 and the exam period largely governing their approach taken to facilitate a timely process.

*‘There was a little bit of me thinking, do I open it up to everyone? But when time was off the essence, and we wanted to get it going, I knew that I, and I’d spoken to these [transition students], I’d said “Look, how do you think? Do you want to be involved?” and they were like “Oh yes please, yes please”, and then, as a result of that initial conversation, because of the type of pupil they are, they were like “What’s happening now with the CHARMING project? Are we doing the CHARMING? Have I been picked?” (SSS02).*

#### Reach and access

Reflective of the targeted recruitment approach, teachers described recruited students as those typically engaged in school and extracurricular activities, in addition to being high performers in physical activities and sports. Peer role models in Cluster 2 described their experience of the selection process, having been approached by a teacher and thereafter answering a series of interview questions. While describing their selection into the programme, the girls expressed that future recruitment efforts should be widened to ensure other students are made aware of the opportunity as they were specifically approached to apply. Similarly, in Cluster 3, peer role models felt that other students might have wanted to be involved and there was a need for wider advertisement. Throughout teacher interviews, there was also a common desire for all eligible students to be given the opportunity to apply for the role.

*I had to go after my creams didn’t I, which is something not necessarily, I would have wanted to do, I probably would have encouraged other pupils that don’t necessarily do much in the community, to be part of this project, because some children do a lot of different activities, be it sport and drama and music. So, I think it would have been an opportunity to tap into those pupils, maybe that are not part of everything. However, like I said, you probably noticed with the girls that you’ve got there, they are some of my best pupils and my best pupils in sport. (SSS01).*

### Research question 2: what are perceived barriers and facilitators to recruiting role models (both community and peer)?

Sixteen community role models were recruited to deliver the intervention, with varying day-to-day roles (e.g., coach, national governing body representative, player-coach, business owner etc.). The barriers and facilitators to the recruitment of role models spanned the themes of communication and logistics.

#### Communication – intervention materials

Overall, community role models viewed the intervention materials positively, with the provided guidance noted as being straightforward. However, some community role models identified areas for improving materials, with a need to add detail of the school premises and equipment availability. Secondary school teachers noted how recruitment instructions and documents were helpful and informative.

*Quantity of information, spot on, information to parents spot on, information to the girls, spot on, did I share much information with the girls? No, because I wanted them to just go over and I said to [CHARMING Primary School Teacher], “We’re coming over and we’re just going to give it a go”, and that’s what I wanted it to be. Have we grown into understanding what the programme is about? Absolutely yes. Would I do it differently? Absolutely yes. What would I do differently? I’d do in assembly. I’d have all my information; I’d probably create a PowerPoint. (SSS02).*

Within Cluster 1, peer role models highlighted the need for clearer and more descriptive detail on what their role entailed. This was seen as particularly important for recruiting students who had no prior experience of being involved in programmes with primary schools. In Cluster 3, the need to advertise the opportunity more widely to create greater awareness of the scheme among other students was also discussed, as peer role models described how other students often queried what they were involved in and why they were leaving school early.

*We were talking about it in our school, because a lot of people were wondering why we were leaving (school early), and they didn’t know about it. They might have wanted to do it. (9PRMFG).*

#### Logistics

Given the COVID-19 pandemic and resultant pressure on schools, the recruitment of community role models was largely coordinated by the research team with support from community stakeholders. Most community role models seemed satisfied with this process, with logistics mirroring their usual practices for engaging with schools and wider groups. As a result, there was minimal ownership by primary schools for community role model recruitment within the current study. To best support schools in their future recruitment activities, community role models suggested the development and use of advertisement to recruit more widely (e.g., creating a job-like description and using social media to disseminate). Similarly, other community role models motioned that an existing organisation such as Sports Wales or local coordinator (e.g., local authority sports teams) would be best placed to coordinate recruitment activities in future.

A barrier to the recruitment of community role models was the need for up-to-date awareness on what clubs and activities exist in the school’s surrounding community. During an interview, one community role model emphasised that while schools are fundamentally the hub of the intervention, they are unlikely to be aware of evolving community groups.

*The only trick is, I don’t have access to the schools, it’s a lot of set up, when you don’t have an in. So, like, if there is a toolkit [listing community activities and clubs available], that would be useful. I would be really interested in trying to do something with that. It could be something that, I almost said something that the schools themselves do, but that will never, I don’t think that will ever work, because they’re too busy already … … perhaps funding gets put in for an entity, to be set up to run it. (CRMI20).*

### Research question 3: are the role model components acceptable to all those involved?

Across all six themes, the involvement of community- and peer role models in the intervention was seen to be acceptable and highly valued by Year 5 girls and all school staff involved. The role models themselves also highlighted several benefits of including both a community- and peer role model element.

#### Interpersonal relationships

Teachers and Year 5 girls described the community role models as knowledgeable, helpful, and supportive. Girls in Cluster 1 reported liking all the coaches that attended, saying they helped teach them more as they were experts. A teacher in Cluster 2 noted the development of relationships and friendships between the primary school girls and community role models, noting the coaching, support, and general encouragement to enhance self-esteem and confidence in performing activities. A secondary school teacher highlighted how the community role models also served as role models for the peer role models, exemplifying careers in sport and showcasing ideas for peers to add to their own coaching repertoire.

*…there was peer role modelling from the person leading as well. So, our lot [peer role models] were looking up to her [Community role model], the little ones [Year 5 girls] were definitely looking up to her … … and I was learning, because she [Community role model] was doing some little drills, and I was thinking, oh my God, I could use that. So, there was that as well, so, they [peer role models] have got a bank of information. (SSS02).*

All teachers emphasised the value of involving former pupils, perceiving peer role models to be more relatable and engaged in the intervention, as they were participating and ‘modelling activities’ alongside the Year 5 girls as opposed to the more instructive role of the community models. In Cluster 3, a teacher also described how this process of going back to the former primary school brought about excitement for peer role models and in one instance, also having the added value of siblings attending the primary school. However, there were some discussions among Year 5 girls about initial feelings of anxiety when around the peer role models, due to a sense of intimidation by their physical stature. However, girls described that these worries abated over time with the connections and relationships becoming more familiar. There was a suggestion that future sessions could include more opportunities to allow communication between the girls and peer role models supporting the familiarisation and friendship building process. Community role models also recalled the positive connections they saw between the girls and peer role models.

*… it’s lovely to see past pupils, you know, where we saw [Peer Role Model], as a school, we were so proud that she came back to the school. She did some additional sessions, and we could then refer to [Peer Role Model], she was a past pupil at [CHARMING Primary School] and then you know, the excitement then obviously was there to be seen. So, it’s lovely to see these, because you know when teachers teach, it’s not half as powerful as having a role model, to deliver the sessions. (1SLT1).*

#### Communication – role clarity

While both roles were deemed highly acceptable, the relationship and role dynamic between the two types of role models was flagged as an area for further refinement. On reflection, community role models and peer role models identified the relatable bridge between their roles. Peer role models supported the idea of acting as a link during the sessions, especially as the beginning of the programme brought high uncertainty over how their role would fit within the programme and the extent to which they would lead or support the delivery within sessions. Both role models identified the need to be clearer on individual roles and expectations and how the roles are set to complement one another.

*We're the step between the leaders and the children. It was quite nice being like that, because we kind of got to do a bit of both, we got to actually enjoy the sport, and go and enjoy it and do it with the kids, but we also got to you know, have a chance of being a leader, things that I'd never done before, so that was quite nice to do. (8PRMFG).*

#### Fit with existing systems

Within Cluster 1, the secondary teacher described how the programme aligned with wider school extracurricular and enrichment practices such as the Welsh Baccalaureate (i.e., an educational qualification focusing on wide ranging skills [43]) and other peer mentoring schemes in primary schools. The CHARMING intervention was viewed as a welcomed opportunity for students to gain experience and support personal and skills development (e.g., undertaking their own risk assessments), with clear contributions towards their curriculum vitae and university aspirations.

Within Cluster 2, the secondary teacher described how the CHARMING intervention had complemented their existing transition work and had also enabled them to engage with a new primary feeder school. This opportunity was perceived as extremely beneficial for the school’s relationship building, resulting in the two schools forging a new programme for the coming year.

Most community role models relayed how the programme was a natural fit with their usual coaching practices and for several, how the programme specifically aligned with their organisation’s aims of increasing awareness and participation of physical activity among girls. In some instances, community role models spoke about the intervention fitting the longer-term visions of the organisation, for example, supporting future physical activity endeavours at university, building avenues to support professional careers in sport and providing development opportunities for coaches.

*I think the club is always really willing to do sessions like this and go and promote because it's not just, I mean you work with kids and you kind of get them into wanting to do it and it promotes us, it promotes the club. We always want more girls to come, we have girls and boys, it's not that, obviously we have both, but I mean let’s be honest, women’s sports in general is always lacking and basketball is not very common in the UK as well. So, the more girls that we can bring in and get them to stay, that's the thing right, like it's getting them to stay, okay you're coming and you're starting the academy and you want them to stay to progress and get them to play hopefully onto a professional level because we have a professional level at the club. (CRM 11).*

#### Perceived impacts

Throughout the data, perceived impacts of the programme underpinned discussions of acceptability, with key areas highlighted below.

#### Forging new connections

School teachers highlighted the opportunities to build relationships between adjoining schools and with local communities. Teachers in Cluster 3 emphasised that they had not previously had a relationship with their secondary school. Furthermore, one teacher spoke about the importance of creating such links with the local community, so girls are made aware of opportunities to continue engaging in physical activities with they learn in physical education, a notable link which the school had often struggled to support girls with.

*I do think, a lot of our girls, they come, they do PE, they might enjoy this sport or that sport and, a lot of the time they’re, like, well, what can I do with it? Where can I go in the community? A lot of the time, like, things aren’t that well-advertised or well known … but, like, the kind of sports that they were getting involved with, I think it’s really good to have that community role model, because it, kind of, it all comes down to we want them in the community and we want everything to have a positive experience in the community. So, I think, you wouldn’t want to get rid of that. I think, they have their role really well regarding the community, then the girls have that really good role of having that, like, peer role model in school, so, you are, like, creating that bubble or where we are between, like, the three links. So, I wouldn’t say it’s negative at all having the community and peer role models involved. (SSS03).*

#### Supporting transition

All schools emphasised the benefit of the programme involving peer role models from adjoining secondary schools. Some suggested the programme could be improved further by giving primary school girls the opportunity to attend some of the sessions at the secondary school. Teachers described how this would support school transition, with the opportunity to become familiar with the new environment and to further learn about physical activity provision at secondary school. Furthermore, the opportunity to meet secondary school staff was also highlighted as a way to build relationships between schools and support transition. Teachers in Cluster 2 also suggested that secondary school-based sessions could be coordinated so girls from other primary schools could also attend the session, enabling girls to forge new relationships ready for school transition in the coming year.

*From a teaching point of view, and from our school point of view, it’s good for transition. It’s good for us to go out, it’s good for them to see children, it’s good for them to work with pupils from [CHARMING Secondary School] and then the conversations start of, “I’m coming up to [CHARMING Secondary School] in September, what’s it like and will you be there?”. Then relationships start to build. (SSS02).*

### Wider perceived impacts

Peer role models described several personal benefits of being involved in the intervention including opportunities to; learn and lead activities, develop communication skills, build confidence, and provide experiences and volunteer time to support future career aspirations. Peer role models appreciated the opportunity to re-visit their former primary schools, with avenues to develop volunteer hours, learn new skills and support future endeavours.

*I think it just gives us confidence, because if we’re starting off with like year fives and year sixes, when we’re older if we want a job to do with like speaking to motivate people, we know what it’s like because we did it, started at a younger age, so it just gives us a bit of an idea of what we’ll be able to do when we’re older. (7PRMFG).*

While not captured during data collection, conversations with secondary school staff post-intervention also revealed continuing positive impacts for peer role models. For instance, in one secondary school, peer role models are continuing to use their role, identifying as ‘the CHARMING squad’ to source and support physical activity opportunities within their own school. 

### Research question 4: what are the perceived barriers and facilitators to implementing the role model components within schools?

Observation data (see Table 1) revealed that the number of peer role models in attendance ranged between 1–8 pupils, with attendance recorded at 81.8% (18/22) of sessions. Findings below draw on observational and qualitative data to highlight four key themes underpinning implementation.

#### Communication – school and community links

A key implementation barrier identified across all schools was a lack of communication. This was described in two situations, firstly between the primary and secondary school cluster (e.g., confirming session start dates and timings) and secondly, communication between schools and community role models (e.g., number of students attending). As a consequence, there were three occasions where no peer role models attended the session; two occasions due to teacher illness and one due a miscommunication of timings. On the contrary, good communication between the schools and research team was identified as a facilitator throughout the programme, with the timely provision of programme timetables and coordination with community role models.

*I’ve just received lots of information which is always helpful. And I’ve always had prior knowledge of who’s coming in, what girls are coming in and if they’re not coming in. (6PST1).*

### Logistics

#### Timing and location of sessions

While some schools highlighted the benefit of after-school sessions, the timing and location of sessions were identified as potential barriers specific to peer role model participation in Cluster 3. Both posed transportation issues between schools while the secondary teacher indicated that travelling to the primary school required a high level of independence, which might limit some students’ ability to be involved. In one school the teacher relayed how they would often assist the peer role models with travel to schools along with wider support from parents.

*When we’re asking them, can you please go to the primary school and then you’re spending your time down there and then get your way home, I do think that then did limit it for some of our kids. So, the ones that did take part the ones where, obviously, it was a lot easier for them to, kind of, make those links down there. (SSS03).*

#### Sharing of information

Observation data revealed three instances whereby the teacher or researcher identified no question-and-answer opportunity within a session. While a clear responsibility of community role models, qualitative data highlighted a lack of time within sessions to carrying this out. Some community role models also described how this was more of an organic process whereby Year 5 girls felt comfortable to ask questions throughout the session rather than needing a dedicated time. Among all clusters it was also evident that peer role models did not engage with sharing information with the Year 5 girls during sessions. Focus group data suggested that this was linked to the peer role models’ lack of role understanding. On occasions, signposting did not take place within sessions and instead, community role models sent materials to the research team for distribution to schools post-session, with COVID-19 limiting the sharing of physical materials. One teacher highlighted how signposting was a highly valued activity, supporting girls to share their experiences at home and identify opportunities for them to continue being active in the community.

*Some of the community role models came with leaflets and handouts, it would be lovely if they all did, because I think, when they go home, sometimes, children are not great at telling their parents what they’ve been doing. So, if they said “What have you done in school today?”, they’d say “Oh I don’t know, ran around outside”, not you know, “Well actually we had a basketball player come in from”, they wouldn’t tell them those things. So, if they had a leaflet, perhaps if they enjoyed it, they’d go home and say to their mums, “This is what we did” or dads, “This is what we did, could I go and join a netball group or a basketball group?” or “Could I take part in taekwondo?”. So, having something physical to give them at the end of the session, almost as a little promotion for them, as well, would be good. (2PST2).*

#### Interpersonal relationships

Observation data showed that overall, peer role models participated in sessions and interacted with the girls, except for one session, wherein peer role models led the session due to community role model absence. Both qualitative and observation data highlighted inconsistencies of peer role model engagement with the intervention across schools. For example, in some instances, community role models and Year 5 girls described the peer role models as unengaged in the sessions yet some of the peer role models said that community role models did not involve them or let them know what they should do. As highlighted earlier, peer role model engagement was described as improving throughout the programme, as time provided an opportunity to build relationships and consolidate their roles.

*Because like it was last week, so obviously they’ve had quite a few weeks already, so they were more comfortable with us. They were asking questions, and because we’re a bit older than them, they could talk to us instead of a teacher, because we can relate more. (7PRMFG).*

#### Fit with existing systems

A key facilitator to implementation was the alignment of the intervention with the schools’ and community role models’ ethos and existing practices. Secondary school staff described how the programme aligned with their aims to provide older girls with opportunities to enhance engagement in physical activity and physical education, with one staff member emphasising this as a prominent area of concern since COVID-19. Several community role models emphasised their passion and drive to make sport a better place for girls and described how this programme provided a vehicle to make that happen. In addition to supporting pupil transition and relationship building between schools, primary school staff described how the programme complemented their focus on pupil health and wellbeing.

*So with us going to these schools as well it can, yes we are delivering the session to keep them active and get them involved in physical activity. But it’s also good for them if they are interested in continuing playing netball they can come over to the development programme that we run over at [Name’s University] which will keep them involved in physical activity. Which I believe is like the main aim of this is keeping them going with physical activity, rather than just finishing school and stop. (CRMI10).*

The totality of data exemplifies that implementation of the role model components was seen as positive with all primary and secondary schools stating that they would be happy to be involved in a future CHARMING intervention. Several suggestions to enhance future implementation were noted throughout the process evaluation with specific recommendations for pre, during and post-programme completion (summarised in Table 2). Recommendations for prior to the programme starting centred on the recruitment of role models (community and peer role models), ownership of community mapping and opportunities to connect peer role models with primary school students. Recommendations for during programme delivery included a consideration of session delivery at secondary school premises and the provision of further information for role models (i.e., role clarity during sessions and guidance on the question-and-answer opportunities). Post-programme delivery, there was a recommendation to provide all participating girls with a certificate of participation.

**Table 2:** Recommendations to enhance future implementation of the role model component

	**Current**	**Future recommendations**
Prior to the programme starting		
Recruitment of community role models	Local authority physical activity teams and publicly available information used to approach role models for involvement	Develop advertisements to recruit more widely (e.g., creating a job-like description and using social media to disseminate) Within information packs add detail on school premises and equipment availability
Recruitment of peer role models	Secondary school teachers were provided with recruitment materials to give to the peer role models	The introduction of a standardised briefing session along with an information pack, would ensure all role models are briefed on the role aims and responsibilities. Consider the use of a training video
Identify community provision	Research team worked closely with local authority physical activity teams to identify community opportunities	Explore the role of peer role models or local authority teams for taking ownership over community activity mapping
Consider travel logistics	No information provided	Explore the option to include location and transport details of feeder primary schools within peer role model information packs
Meet and greet opportunity	Intervention materials encouraged peers to introduce themselves to the Year 5 girls at the start of the session	Provide an opportunity for the peer role models to meet the Year 5 girls ahead of the intervention starting The information pack to contain greater detail on the “meet and greet role” emphasising key information for sharing with the Year 5 girls and approach to weekly introductions with community role models
During the programme		
Session venue	Sessions took place on the primary school premises	Explore options for some sessions to take place on the secondary school premises. Including a meet and greet with the physical activity lead teacher at the Secondary school
Dynamic between community and peer role models	No information provided	Information pack to provide clear guidance on the differentiation of roles and how these can complement one another in practice
Concluding the sessions	Peer role models to share insights about physical activity at secondary school during question-and-answer opportunity	Explicit guidance to be added to the information pack on the types of information to share e.g. Physical Education lessons, after-school clubs, community clubs
Post programme completion		
Programme recognition	Certificates for peer role models sent to secondary school	Use of a certificate ceremony to congratulate all girls on taking part in the programme, with recognition from both primary- and secondary schools

## Discussion

This paper reports process evaluation findings related to the role model components of the CHARMING intervention. Drawing on experiences of role models, teachers and 9–10-year-old girls participating in the CHARMING intervention and observational data, findings demonstrate that overall, the inclusion of community- and peer role models in the programme is both acceptable and feasible.

The inclusion of peer role models from adjoining schools, within the CHARMING intervention was an element informed by earlier development work [37], therefore prior knowledge of the logistical considerations for sourcing role models was lacking. Study findings highlight that it is possible to source peer role models from adjoining secondary schools and furthermore, that there is a clear desire for the intervention to span both the primary and secondary school, with a wider aim to support pupil transition across settings. While findings demonstrated modifications to recruitment approaches due to COVID-19 (i.e., research team coordinating community role model recruitment and secondary school teachers taking a targeted recruitment approach as opposed to inviting all eligible students), teachers were confident with their ability to recruit students for the role and students recognised benefits from being involved in the programme. Communication was identified as a facilitator to the recruitment of both community and peer role models, with largely positive accounts of recruitment materials and communications with the study team. Regarding the logistics of future coordination of community role model recruitment, findings suggested that ownership could be best placed with local physical activity providers as opposed to schools. This remains a key area of work to be explored within future efforts to optimise the intervention and identify an approach to implementation that would be replicable outside of the context of a research study.

Evidently, the fit of the CHARMING intervention with existing systems and structures was a key facilitator to intervention acceptability and implementation. Both school staff and community role models spoke of the complementary nature of the programme to existing activities and for some schools, how it had encouraged needed community links. The use of school-community partnerships, whereby schools extend their traditional focus beyond education to include health and involve the wider community [44], has been shown in the field of physical activity promotion whereby schools and communities join forces to provide more physical activity opportunities for students [45]. Underpinned by theoretical frameworks [46], wider research [47] and policy recognitions [6, 48], partnerships offer the opportunity to harness the existing expertise and physical activity infrastructure available within the local community. Van Acker and colleagues [45] stress the need to construct local strategic partnerships which span across multiple communities to support programme sustainability and offer local tailoring to school and community contexts.

Throughout the data, evident areas of contention were the role dynamic and role distinction between the two types of role models. The lack of role clarity was reflected in observational data whereby peer role models were consistently shown to not contribute information within sessions, while on some occasions community role models did not provide question-and-answer opportunities. To facilitate future delivery, the findings suggest there is a need to further develop the details included within programme materials to ensure role models are clear on their own role and responsibilities and to shed light on the complementary role model’s role. While wider research [49, 50] has shown that coaches can often rely on their prior knowledge to deliver sessions as opposed to following intervention materials, it is important that community role models are aware of the importance of planning adequate time to deliver all core elements within each session.

Throughout participant accounts of intervention acceptability, there was a strong focus on perceived impacts of the programme. Interestingly, perceptions concerned impacts beyond the central focus of increasing physical activity, with accounts of how the intervention could support transition and connections between schools, benefit peer role models and support wider ripple effects into secondary school settings.

### Recommendations for future role model programmes and policies

- Identify a key contact who is responsible for the recruitment of role models.
- Provide guidance and allow sufficient time to support an inclusive approach to role mode recruitment.
- Consider how and when role models might be introduced to participants within a programme.
- If different types of role models are included, consider programme roles and dynamics between role models and what specific guidance might be required for each.
- Consider what recognition role models may desire for participating in a programme.

#### Strengths and limitations

Strengths of this study involve the comprehensive sampling approach taken to ensure diversity of perspectives across a range of participants, with all role models being given the opportunity to participate. Furthermore, the thorough approach to data analyses aimed to strengthen rigor and transparency throughout the study. This study has several limitations. First, COVID-19 influenced the recruitment approaches for community role models and in some schools, the recruitment of peer role models, which warrants additional future work to explore this in a non-pandemic context. Second, it would have been desirable to collect information from parents particularly regarding the logistics of travel to and from sessions. Third, a large proportion of observation data were missing from teacher observation sheets which limited our ability to fully explore certain areas of acceptability and feasibility within qualitative discussions. Fourth, it is important to acknowledge the possibility of social desirability bias when interpreting the study findings. Fifth, the composition of girls’ focus groups was largely determined by the lead teacher, with some teachers present throughout the focus group and others absent. Subsequently we are limited in the selection of students and variation in the conduct of data collection. Furthermore, it’s important to note that the duration of interviews and focus groups varied considerably, with some participants limited by time availability and others less forthcoming within discussions.

## Conclusions

Findings of the current study provide an in-depth understanding into the role model components of a school-based, community linked intervention, highlighting both contextual and dynamic factors influencing implementation. Showcasing coordinated efforts across multiple parts of the school-community system, findings reveal the future needs of the programme, with clear areas for improvement and further optimisation. This information can also be used to inform wider interventions or policies employing several role models across settings to promote physical activity among children.

### Supplementary Information

**Additional file 1.** CHARMING Intervention logic model.

## Data Availability

The datasets generated during the current study are not currently publicly available due to wider publication plans but are available from the corresponding author on reasonable request.
