# Supplementary material for: A mixed-methods process evaluation of the feasibility and acceptability of involving community and peer role models within a physical activity intervention for primary-school-aged girls (the CHARMING study)
Source: BMC Public Health. 2023 Oct 7;23:1950. doi: 10.1186/s12889-023-16826-x (PMC10560422; doi:10.1186/s12889-023-16826-x)
Supplement: Supplementary file 1 — Additional file 1. CHARMING Intervention logic model. [file 12889_2023_16826_MOESM1_ESM.docx]

**CHARMING Intervention logic model**

SHORT-TERM OUTCOMES

**Cognitive processes**

- Increased awareness of community physical activity opportunities
- Developed an interest in being more physically active

**Intermediate**

- Increased enjoyment of physical activity
- Improved self-efficacy, attitudes, skills and knowledge relating to physical activity
- Increased autonomy
- Facilitate school-transition
- Peer role models from adjoining secondary school
- 1-hour weekly physical activity taster sessions (after-school for 1 term)

- Community role model delivering the session

- Peer role models participating in the session
- Post-session Q&A session with role models
- Signposting to community activities and clubs

OUTCOMES

- School-community partnerships
- Mapping of community physical activity provision
- Transport options

PLANNED COMPONENTS

INPUTS

- Community role models
- Appealing activities
- Equipment and space on school premises

**Behavioural processes**

- Increase socialisation and relatedness with peers and role models
- Increased confidence, competence, and self-efficacy
- Experience of new physical activity/sport
- Intention to take up new activity/join club
- Increased physical activity levels

**Long-term**

- Frequent attendance at a community club/activity
- Sustained physical activity
- Improved children’s fitness
- Reduced sedentary time

**Displacement**

- Does intervention attendance replace other physical activity?
